# The National Insurance Bill and the Voluntary Hospitals

**Published:** 1911-05-27

**Authors:** Walter G. Carnt

**Affiliations:** General Superintendent and Secretary, Manchester Royal Infirmary.


### STATE INSURANCE.

### THE NATIONAL INSURANCE BILL AND THE VOLUNTARY HOSPITALS.

### HOW IT WILL AFFECT THE WORKERS.

By WALTER G. CARNT, General Superintendent and Secretary, Manchester Royal Infirmary.

What will be the effect of the National Insurance Bill upon the voluntary hospitals? When the Chancellor of the Exchequer brought in the Bill in the House of Commons, his speech was a masterly exposition of the details of a scheme as far-reaching as, if not more far-reaching than, any proposals laid before the House during the lifetime of the present members. The principles of the Bill received a cordial welcome from all parties in the House, and the impression created throughout the country as indicated by the Press was one of almost unqualified approval.

The Hospitals.

The administrators of voluntary hospitals, who are day by day brought into touch with much suffering, who hear many pitiful tales of pinching poverty during the period the breadwinner of a family is an inmate of a hospital, and who have frequently longed for a large surplus income, or a huge Samaritan fund to enable a helping hand to be extended to those who, in addition to suffering intense anxiety in consequence of the serious condition of the husband or father, have the harassing knowledge that there is little or no income entering the house and there is little or nothing put by for a rainy day, read the details of the scheme with a thankful spirit because the weekly payments to be provided during sickness would enable the wolf of abject poverty to be excluded from many households. As they read on, that feeling of thankfulness became mixed with a feeling of disappointment, because they failed to discover what they naturally expected to find—namely, a reference to the voluntary hospitals which have been finding for the last 150 to 200 years (and in some instances 400 or 500 years) the medical treatment for a large majority of those for whom provision is now to be made by the State. The benevolent founders and supporters of these institutions have (frequently at the expense of much self-sacrifice) supplied year by year the huge income needed to treat those who without their aid must have died prematurely or become chronic invalids or maimed helpless individuals, a burden to themselves, to their relatives, or to the rates.

Notable Omissions.

An examination of the Bill explains the lack of reference to hospitals in the Chancellor's speech. There is no reference to them or practically none in the Bill itself, and apparently they form no part of the Chancellor's scheme for the amelioration of the lot of the sick poor. So far as can be discovered by a careful perusal of the Bill, the existence of hospitals is referred to only thrice, although by a stretch of the imagination they may possibly be intended to be referred to in several clauses. The following are all the clauses which may be so construed:

Clause 8.—The benefits conferred by this part of this Act upon insured persons are: (*b*) Treatment in sanatoriums *or other institutions when suffering from tuberculosis or such other diseases* as the Local Government Board with the approval of the Treasury may appoint (in this Act called sanatorium benefit).

Clause 12.—1. No payment shall be made in respect of sickness, disablement, or maternity benefit to any insured person during any period when he is an inmate of any workhouse, hospital, asylum, or infirmary, supported by any public authority or out of any public fund or *by a charity*, or of a sanatorium or similar institution established under this part of this Act. 2. During such period as aforesaid any such benefit which would otherwise have been payable to such person, (*a*) Shall be paid to or applied in whole or in part for the relief or maintenance of his dependants (if any) in such manner as the society or committee by which the benefit is administered thinks fit; or (*b*) If such person is an inmate of a sanatorium or similar institution in which he is receiving treatment in accordance with the provisions of this part of this Act and has no dependants, shall be paid to the Local Health Committee towards the general purposes thereof. 4. The society or committee by which the sickness, disablement, or maternity benefit of any insured persons is administered may later enter into any agreement with the proper officers or authorities of any convalescent home or sanatorium, admission to which is conditional on the payment of the whole or any part not less than one half of the cost of maintenance, for the payment thereto of any sickness, disablement, or maternity benefit which would, apart from this section be payable to, or in respect of, any such person who becomes an inmate of such home during any period for which he remains an inmate.

Clause 13 (part of).—Sanatorium benefit shall in all cases be administered by and through Local Health Committees.

Clause 15.—1. For the purpose of administering sanatorium benefit Local Health Committees shall make arrangements to the satisfaction of the Insurance Commissioners, with persons or local authorities having the management of sanatoria or other similar institutions approved by the Local Government Board, for the treatment therein of insured persons entitled to sanatorium benefit under this part of this Act.

Clause 17.—It shall be lawful for an approved society to grant such subscriptions or donations as it may think fit to hospitals and other charitable institutions, or for the support of district nurses, and to appoint nurses for the purpose of visiting insured persons who are members of the society, and any sums so expended shall be treated as expenditure on such benefits under this part of this Act as may be prescribed.

The Friendly Societies.

A casual reader would almost gather from the Chancellor's speech that the Friendly Societies were the only agencies which have ameliorated the lot of the sick poor of this country, when, as a matter of fact, those great societies which have done so much to promote thrift and to create and preserve in the minds of the working classes a spirit of self-reliance and self-help, have in reality only touched the fringe of the sickness question, so far as treatment is concerned, the actual amelioration of the lot of the sick poor above the pauper class having been carried out by the voluntary hospitals, the provident dispensaries, private sick clubs of members of the medical profession, district nurses' associations, etc.; and this being so it seems an anomaly that the control of State-aided medical treatment should, by the provisions of the Bill, be handed over almost exclusively to the Friendly Societies.

### The Annual Subscription List.

The outlook of the voluntary hospitals, unless the Bill can be radically changed before it becomes law, is a serious one. They have always had to struggle to make both ends meet, but somehow, in spite of vicissitudes and adverse balances, they have kept going and made wonderful strides. The legislation of recent years has made the struggle rather more arduous, but no previous legislation has created in the minds of hospital administrators the dismay that is taking possession of them the more the provisions of the Insurance Bill are studied. The backbone of the income of most of the voluntary hospitals is the annual subscription list. It has always been a difficult matter to increase it, and in these hard times there is no concealing the fact that a large proportion of these subscriptions are given by employers because the hospital is the chief helper of their workpeople in sickness and after accident, and so far as sickness is concerned the employers seldom make other payments, or perhaps it would be more correct to say comparatively seldom. They will now be saddled with an enormous annual charge which is to provide for medical attendance, medicine, and a sick allowance for their employees. Are they likely to be willing or to be able to afford to pay a voluntary subscription to the hospital? The answer must be in the negative, or at least it appears at present that this must be so. A very substantial portion of the income of a hospital is derived from the contributions of the workers themselves. Will they be able to afford to contribute to hospitals after they have paid the heavy weekly payment for insurance? It is to be feared that the answer must again be in the negative. The effect of the Bill will probably be the same in practically every source of the hospital income—smaller church collections, fewer and smaller donations and legacies. The outlook is indeed discouraging.

It may very fairly be asked if the hospitals will derive any compensating advantages from the wide distribution of medical benefit. There is little doubt that the work will be reduced. The worker will no longer be eligible as an out-patient—he or she can obtain medical treatment elsewhere—but the dependants of the majority of the poorer workers will still flock to the hospital. Regular wages will remain the same or may possibly be slightly lower. If the worker could not afford to pay for medical attendance for his wife and family before the Insurance Bill existed, he will be less able to pay for it after it has become law and he has heavy weekly payments to make. The number of out-patients will therefore be reduced possibly by one-fourth. Out-patients are, of course, the least expensive of the persons treated at hospitals, therefore the reduction of expenditure will not be very great.

It is difficult to foresee how the out-patients in special departments are to be reduced; the sum to be allowed by the State for the payment of the doctor is too meagre for the general practitioner. It cannot possibly satisfy the specialist in diseases of the eye, ear, and throat, and of women. Where will the worker get this assistance if not at the voluntary hospital?

With regard to in-patients, it would appear that very little diminution in numbers is likely to arise; the workers have no suitable accommodation in their houses for the treatment of a person seriously ill or in need of operation. Such cases must come to the voluntary hospital, and there is little doubt that the wards will be as full as ever.

It must be remembered, too, that the hospital as an employer of labour will have to contribute to the insurance scheme, although it invariably treats its employees during illness and frequently pays the salary or wages in addition.

### The Problem for the Hospitals.

The conclusion to be arrived at appears to be therefore that the hospitals can expect to have only slight reductions in expenditure, and the problem for solution is, Where are the funds necessary for their maintenance to be obtained?

Can hospital authorities rely upon the Local Government Board with the approval of the Treasury construing the ailments of patients in general hospitals as the "other diseases" referred to in Clause 8 (*b*) and the general hospitals themselves as the "other institutions" referred to in the same clause? It appears exceedingly doubtful.

Clause 12 (1) removes the possibility of the patient being in a position to pay to the hospital the sum he would receive for maintenance if he remained at home, as it expressly lays down that it shall not be paid to him during the period he is an inmate of a charity; and

Clause 12 (2) (*a*) devotes this sum to the maintenance of his dependants (if any), which is only right.

Clause 12 (2) (*b*) states that if he has no dependants it is to be paid to the Local Health Committee.

If Clause 12 (4) can possibly be construed into including hospitals, it would appear that there is a possibility of entering into an agreement for payment with the society or committee which administers the "sickness" or "disablement" benefit.

If Clause 15 (1) can be construed into including hospitals, an agreement for payment with the Local Health Committee may be possible.

Clause 17 makes it lawful for an approved Friendly Society to give subscriptions and donations to hospitals.

With the exception of Clause 17 there is nothing really tangible from a hospital point of view in these provisions, and knowing, as all hospital administrators do, how comparatively few are the Friendly Societies which subscribe to hospitals, although they have the power to do so to a small extent out of their "management funds" if they are so disposed, the prospect of obtaining really substantial help from these societies is not very hopeful.

### Sweating the Staff.

There is also the serious question of the honorary medical staffs. They have given lavishly of their skill and time for the benefit of the poor who have sought the aid of the voluntary hospitals. Now that the State proposes to undertake the responsibility of the care of the workers during sickness, can the State expect the gratuitous services of the highly skilled consultants and others who form the honorary medical staffs of these institutions? The State has no right to expect anything of the sort, but as no reference has been made in the Bill to the hospital treatment of any patient or how the work of such institutions is to be adapted to the new order of things it is impossible to say what is intended.

It has been the pride of most voluntary hospital administrators that the financial support has been "voluntary." The assistance rendered by municipalities or Boards of Guardians has been infinitesimal compared with the total income, and could not, as such assistance have been voluntary grants, detract in any way from the "voluntary" ideal, but this Insurance Bill appears to indicate that we have now come to the parting of the ways. The voluntary income will undoubtedly be reduced. There will be no corresponding reduction of expenditure. The State by its legislation will be responsible for the reduction. Voluntary hospitals should look to the State to replace the deficiency.

### State Aid for the Hospitals.

The State contributes large sums to Universities, leaving their control in the hands of the University authorities. In Australia and other British dominions the hospitals are subsidised by the State without the State assuming direct control. Why not a similar arrangement in England?

It would appear, therefore, that hospital authorities should spare no effort to ensure that the Bill be so amended that there is no ambiguity as to the position of the voluntary hospitals. They cannot be ignored in any scheme of national sickness insurance. They have borne the brunt of the work hitherto and they will continue to carry out the major portion of their previous duties, and provision should be made in the Bill for adequate contributions to be made by the State to enable them to be maintained in the high state of efficiency which now exists.

The British Hospitals Association should convene a meeting in London to consider the Bill from the voluntary hospital point of view and invite every voluntary hospital in the country to send representatives, with the object of being in a position to forward to the Chancellor of the Exchequer an authoritative pronouncement on the subject.

